# Effect of a brief intervention on evidence-based medicine skills of pediatric residents

**DOI:** 10.1186/1472-6920-6-1

**Published:** 2006-01-10

**Authors:** Eugene Dinkevich, Andrea Markinson, Sama Ahsan, Barbara Lawrence

**Affiliations:** 1 Department of Pediatrics, SUNY-Downstate College of Medicine, 450 Clarkson Avenue, Brooklyn, NY, USA; 2 Institute for Evidence-Based Practice, Medical Research Library of Brooklyn, SUNY-Downstate College of Medicine, 450 Clarkson Avenue, Brooklyn, NY, USA; 3 SUNY-Downstate College of Medicine, 450 Clarkson Avenue, Brooklyn, NY, USA

## Abstract

**Background:**

While Evidence-Based Medicine (EBM) skills are increasingly being taught in medical schools, teaching quality has been insufficient, so that incoming pediatric residents lack adequate EBM skills required for patient care. The objective of this study was to evaluate the effectiveness of a brief teaching module developed to improve EBM skills of pediatric residents.

**Methods:**

With-in subjects study design with pre- and post-test evaluation was performed in a large urban pediatric residency training program in Brooklyn, New York. We included PGY-1s during intern orientation, while second and third year pediatric residents were selected based on schedule availability. Sixty-nine residents were enrolled into the study, 60 (87%) completed the training. An EBM training module consisting of three or four weekly two-hour seminars was conducted. The module was designed to teach core EBM skills including (1) formulating answerable clinical questions, (2) searching the evidence, (3) critical appraisal skills including validity and applicability, and (4) understanding levels of evidence and quantitative results for therapy articles. A portion of the Fresno test of competence in EBM was used to assess EBM skills. The test presented a clinical scenario that was followed by nine short answer questions. One to three questions were used to assess EBM skills for each of the four core skills. The κ co-efficient for inter-rater reliability was 0.74 (95% CI: 0.56–0.92).

**Results:**

Prior to the training module, the residents achieved a mean score of 17% correct overall. Post intervention, the mean score increased to 63% with improvement in each EBM category. A mean of 4.08 more questions (out of 9) were answered correctly after the training (95% CI of 3.44–4.72).

**Conclusion:**

A brief training module was effective in improving EBM skills of pediatric residents.

## Background

Training in Evidence-Based Medicine (EBM) Skills has been widely implemented throughout medical school and residency curricula. The quality of training that medical students receive in the US has not been consistent, while residents who attended medical school outside the US may not have received adequate EBM training. A recent randomized study done in the US found that medical students were not able to adequately use quality of evidence to guide clinical practice[1]. Despite 95% of Internal Medicine residencies reporting having journal clubs and 37% having freestanding EBM curricula, few studies have looked at the effectiveness of EBM training[2]. This deficit has been ascribed to logistical difficulties such as small sample sizes, frequent rotations of residents, and limited time allotted for EBM training[3]. In addition, until recently no validated measures of EBM skills were available, forcing researchers to rely on variables such as satisfaction and self-reported changes in attitudes. These variables are subjective and inadequate measures of learners' knowledge [4]. As a result, most studies have measured attitudes toward practicing EBM rather than actual skills. Many of the studies that attempt to measure actual EBM skills have focused on teaching appraisal skills but not the other core competencies of EBM practice [5,6]. More recently, validated instruments of core EBM skills have been developed that allow investigators to measure EBM competencies[3,7].

The problem of lack of evidence to support EBM training has been particularly obvious in the training of pediatricians. There are fewer evidence-based pediatric resources available when compared to resources available for internists, and fewer studies examining the practice of EBM during pediatric residency training [8]. To address these problems, the authors of this study developed a brief EBM teaching module. The objective of the module is to provide pediatric residents with basic EBM skills that would serve as a foundation to be built upon during residency training and didactic conferences including EBM morning report and EBM journal club. This module focused on four core EBM skills of (1) formulating clinical questions, (2) searching the evidence, (3) understanding levels of evidence and quantitative results for therapy articles, (4) and critical appraisal skills including validity and applicability[9]. The primary objective of the study was to show whether a brief training module could provide residents with basic EBM skills.

## Methods

### Study design

A within-subjects study design with pre- and post-test evaluations of residents' EBM skills was used. Pediatric residents at a large, university-based hospital, in Brooklyn, NY, participated in the study. This institution serves an urban population and attracts both United States and internationally trained physicians for residency training. Approval for collecting data and reporting results was obtained from the institutional review board prior to initiating the study.

### Educational intervention

Initially, the EBM training course consisted of four two-hour sessions held over four weeks. The course was modeled after the EBM training program developed by the McMaster University in Canada[10]. Each session was followed by a homework assignment. In the first session, the residents were introduced to the concept of EBM and how it is used in clinical practice. They were taught to recognize situations that involve clinical uncertainty and whether their information needs constitute a background or foreground question. The former, such as a question about etiology or pathophysiology of a condition or a disease, may be searched in a paper or electronic textbook. The students learned that EBM skills are helpful in answering foreground questions, including questions about therapy, diagnosis, prognosis, or harm. From this point on the seminar focused only on questions related to therapy. Students were taught to formulate focused clinical questions. In addition, this session covered levels of evidence and introduced students to various EBM resources available at our institution. As a homework assignment, residents were given a clinical scenario and asked to form focused clinical questions and perform a search for evidence.

The second session was dedicated to developing searching skills and improving searching efficiency. Residents were introduced to Web-based EBM resources available within and outside the institution, including the institution's EBM tutorial [11] and the Evidence-Based Medicine Toolkit from the University of Alberta (Canada) [12]. They were taught when to use primary, secondary and tertiary EBM resources, and instructed on efficient MEDLINE use. The homework assignment required learners to appraise a randomized control trial of amoxicillin vs. placebo for the treatment of acute otitis media using the EBM toolkit[13].

The third session reviewed measures of difference for therapy questions including: (1) absolute risk reduction, (2) relative risk reduction, (3) number needed to treat, and (4) interpretation of confidence intervals. In addition, the residents learned how to determine whether study results are valid and applicable to the patient in question. This session was based on the JAMA's "Users' Guides to Medical Literature" series [14] and the EBM Toolkit. The homework assignment required a synthesis of the curriculum thus far: selecting a patient, formulating a focused clinical question, searching the evidence, and appraising an article with respect to validity and applicability to their patient.

In the fourth session, residents were asked to discuss their searches and appraisals. It was also an opportunity to ask questions and review. Residents were also asked to complete the post-test evaluation at the end of this session.

A preliminary review of the effectiveness was conducted after approximately 40 residents completed the training module. At that time, the training was reduced to three sessions. This decision was based on the resident time constraints and the apparent effectiveness of the four-session training module. When the training was reduced to three two-hour sessions, the concepts taught remained unchanged but information was consolidated. In addition to the introduction to EBM, typology of focused clinical questions, and levels of evidence, the first session covered the basics of MEDLINE searching. Strategies to improve searching efficiency opened the second training session that now also included training in measures of difference for therapy questions. The third session covered article appraisal and questions from the learners were answered. The post-test evaluation was administered at the end of the third session.

### Participants

Training was offered to all incoming PGY-1's during the orientation session prior to the beginning of internship. Second and third year pediatric residents received training during a four week period before EBM morning report and EBM journal club were instituted at the residency program. Of the 69 residents enrolled into the study, 60 (87%) completed the training. The other nine residents who did not complete the training withdrew from the seminar due to changes in rotation schedules or illness that precluded attendance at the weekly sessions. Sixty nine percent of the residents who completed the training were PGY-1, 15% were PGY-2 and 17% were PGY-3. Fifty three percent were 25–30 years old, 43% were female, and 35% were US medical school graduates. The first 42 residents (70%) received four two-hour sessions, while the rest received three two-hour sessions.

### Outcome measures

A written test of EBM skills was employed as the primary outcome measure. The Fresno test of EBM competence, [6] a validated test of competence in evidence-based medicine, was employed to assess EBM skills taught in the training module. The Fresno test includes two clinical scenarios that suggest clinical uncertainty. Residents were asked to choose one scenario and respond to short-answer questions on EBM skills necessary to manage the patient in the scenario. The test took 30–45 minutes to complete. Nine questions from the Fresno test were used to evaluate the four core EBM skills. (The three questions on articles about diagnosis and prevention were not used because those skills were not taught in this training module.) The ability to formulate clinical questions was evaluated by one question. Finding the evidence was measured by three questions. Critical appraisal skills were evaluated by two questions. Understanding measurement of therapeutic difference was measured by three questions.

A standardized grading system using explicit grading criteria was used to evaluate residents' performance. Since the objective of the brief training module was to teach basic EBM skills, the grading system of the Fresno test was adapted to measure achievement of basic EBM skills only. Answers were assigned a score of 0 (inadequate skill) or 1 (adequate skill). For example, to answer the first question the resident had to write a focused clinical question. To receive a score of 1, residents needed to include the four key components of a focused clinical question: patient population, an intervention, a comparison group and a measurable outcome. Scores from individual questions were added to determine summary score of EBM skills. One of the authors (SA) graded all of the questionnaires. The grader was blinded to the identity of the resident and whether the questionnaire was given before or after the training. Another author (ED), similarly blinded, regraded 10% of the sample. The κ co-efficient for inter-rater reliability was 0.74 (95% CI: 0.56–0.92) using agreement on scoring of answers to each question as a base. For individual core EBM skills, the κ coefficients were as follows: Formulating clinical questions, 0.69; Finding the evidence, 0.79; Appraising validity, 0.74; and Understanding measures of difference, 0.76.

### Statistical methods

A within-subjects study design with pre- and post-test evaluations of residents' EBM skills was used. A within-subjects *t*-test was used to test for the significance of change. An analysis of variance was used to test the effect of the number of training sessions.

## Results

Before the training module, the residents achieved a mean score of 17% correct overall, showing that, on the average, they had little knowledge of EBM skills. Post intervention, the mean percentage of questions answered correctly increased to 63% with significant improvement in each EBM category. Using a difference score as the criterion in a within-subject *t*-test, the mean difference between pre- and post-test number of questions answered correctly was 4.08 out of 9.00, a statistically significant result with a 95% CI of 3.44–4.72 (*p *< 0.0001). Table 1 shows the test results for the pre- and post-tests for each EBM category. There was a small but significant difference (*p *< 0.04 using an analysis of variance) between students who received the training in three sessions and those who received four. The mean increase for those who received three sessions was 3.63 questions answered correctly; for those who received four, 5.05 questions.

## Discussion

The results of this study show that a three or four sessions brief educational module can significantly increase residents' knowledge of the core EBM skills.

Previous research has demonstrated that core EBM skills can be taught effectively to residents. Green et al. used an EBM curriculum based on adult learning theory to demonstrate that a 7-week EBM curriculum improved skills of Internal Medicine Residents.[15] Smith et al. demonstrated a similar result after a 14-hour intervention for all core EBM skills except critical appraisal [16]. A recent systematic review of 23 studies in EBM training demonstrated that resident EBM skills are much more likely to improve when the duration of training was at least eight hours, regardless of whether the training was integrated into a larger EBM curriculum[17]. Most of the studies that evaluate training residents in EBM have been done in internal medicine and family practice residents. Little information is known about EBM skills of pediatric residents. In fact, an extensive search of both MEDLINE and the Internet found no reported evidence of any other EBM seminar programs with pre-post testing in pediatric residency programs. There is one report of an EBM journal club done in a pediatric residency where only a limited self-assessment was performed [18].

This study demonstrates that significant gains in skills can occur even with a very brief intervention of six hours administered during resident orientation. This is an important point, particularly in a busy pediatric residency training program where heavy clinical responsibilities make it difficult for residents to attend didactic conferences, especially consecutive seminars that build on one another. Residents will be able to use these skills as a foundation to develop and build upon during their clinical training including inpatient and outpatient rotations as well as didactic seminars such as EBM morning report and the EBM journal club.

Previous research has demonstrated that an integrated approach to EBM skills training, where EBM skills are integrated with patient care through the attending rounds and/or cases taken from resident's actual patient care, results in better acquisition of EBM skills than the didactic, lecture based approach [17]. This study attempted to simulate an integrated approach by asking the trainees to come up with questions that were based on real patients, and using questions generated by other residents. This was not always possible, however, especially when the training sessions took place before the beginning of the the academic year.

This study has a number of limitations. First, residents were not randomized to receive the EBM training, but rather served as their own controls in an quasi-experimental study design. This study design is commonly used to evaluate the effectiveness of residency curricula because it is least likely to interfere with clinical work performed by the residents and maximized the likelihood of residents attending all four seminars[19]. All PGY-1's received training during the orientation sessions and before starting clinical work. Since no other didactic training took place during this time, it is unlikely that improvements in EBM skills was due to anything but the training module. PGY-2's and 3's received EBM training during a 4 week period prior to institution of EBM morning report or journal club. It is also unlikely that score improvements in these residents were due to factors other than the intervention, particularly in light of the large magnitude of score improvements and absence of any other EBM training.

Second, while a validated measure of core EBM skills was used, it was modified for use in this study. The original Fresno test of EBM competence measures four levels of competency in EBM: excellent, strong, limited, and none. The objective of this study was to measure achievement of only the basic EBM skills, therefore the grading system was changed to measure only adequate and inadequate skill. It was also necessary to modify the test to better reflect the curriculum of the intervention. The original Fresno test evaluates the EBM skill for both the Therapy and the Diagnosis questions. Since our training module only taught Therapy skills, Diagnosis questions were omitted. In addition, the residents took essentially the same test, but with a different clinical scenario. Some of the improvement in scores may have been the result of residents' learning to answer the same questions better, but the marked degree of improvement suggests that substantial generalizable learning took place.

Finally, the study sample size of 60 residents is a limited sample that may not be reflective of all the pediatric residents in the US. It is however, a relatively large sample for studies of resident performance.

## Conclusion

This study demonstrated that a very brief training module was effective in improving knowledge of EBM skills as measured by the difference between the pre and post test scores. Future research needs to focus on whether these skills are practiced during residency training and on their impact on patient care.

## Competing interests

The author(s) declare that they have no competing interests.

## Authors' contributions

Eugene Dinkevich conceived the study, participated in the design, administered the intervention and drafted the manuscript, Andrea Markinson participated in the design, administered the intervention and drafted the manuscript. Sama Ahsan participated in the design, data collection and drafting the manuscript. Barbara Lawrence participated in the study design, data analysis and drafting of the manuscript. All authors read and approved the final manuscript.

## Pre-publication history

The pre-publication history for this paper can be accessed here:



## Figures and Tables

**Table 1:** Resident performance on the Fresno test of EBM competence before and after the brief training module

Category	Percent Correct		*p*-value
	
	Pre-test	Post-test	
Formulating answerable clinical questions	5%	80%	0.0001
Finding the evidence	22%	53%	0.0001
Critically appraising the evidence	27%	71%	0.0001
Measuring difference between groups for therapy articles	11%	61%	0.0001
Total	17%	63%	0.0001
